# Report on Rare Complication Post Silent Myocardial Infarction: Ventricular Septal Rupture

**DOI:** 10.7759/cureus.37389

**Published:** 2023-04-10

**Authors:** Luna Thapa, Lucas Lemons

**Affiliations:** 1 Emergency Department, West Virginia School of Osteopathic Medicine, Lewisburg, USA; 2 Emergency Medicine, King's Daughters Hospital, Ashland, USA

**Keywords:** reperfusion, shortness of breath, heart failure, ventricular septal defect, ventricular septal rupture, myocardial infarction

## Abstract

The advent of primary reperfusion therapy for the treatment of myocardial infarction (MI) has made mechanical complications rare. Mechanical complications include free wall rupture, papillary muscle rupture, left ventricular septal rupture, and more. In this case, we describe a 53-year-old patient who presented to the emergency department with complaints of shortness of breath, abdominal pain, urinary retention, and constipation. On exam, he was in mild distress and presented with jugular venous distension (JVD), bibasilar crackles, and diffuse abdominal pain with guarding. After a rapid hemodynamic decline and a transthoracic echocardiogram that displayed a new onset ventricular septal defect (VSD), it was determined that the patient had a ventricular septal rupture (VSR). Septal rupture is a cardiac emergency causing cardiogenic shock and carries a high mortality risk despite prompt surgical treatment; hence a high suspicion is warranted. Our patient presented with generalized symptoms, no previous cardiovascular history, and no reported myocardial infarctions or risk factors, leading to a low clinical index of suspicion for a VSR. This case highlights the importance of high clinical suspicion of ventricular septal rupture in a patient presenting with similar symptoms so prompt management can occur.

## Introduction

Mechanical complications after a myocardial infarction (MI) are infrequent in the current era due to the advancements in catheter-based, surgical, and pharmacological reperfusion techniques. However, they occur in patients with large or undetected myocardial infarctions where revascularization did not occur. Some common mechanical complications include papillary muscle rupture, free wall rupture, and ventricular septal rupture [1]. These complications have extremely high fatality rates, so immediate diagnosis and surgery are critical.

This article presents a patient who arrived at the hospital with complaints of shortness of breath, worsening abdominal pain, diaphoresis, urinary retention, and constipation and was determined to have a ventricular septal rupture. This case was noteworthy because this patient had no previous cardiovascular history and no reported recent myocardial infarction, which prolonged the diagnosis of ventricular septal rupture.

## Case presentation

A 52-year-old male with a past medical history of chronic pain syndrome, rheumatoid arthritis, and seizures presented to the emergency department with complaints of orthopnea and worsening abdominal pain that began 3 days ago. He also complained of nausea, urinary retention, and constipation. The patient denied any history of hypertension, diabetes, hyperlipidemia, congestive heart failure, myocardial infarction, or other pertinent medical histories. He did admit to a 39-pack-year history. His family history included heart issues in his dad and grandfather, which he denied knowing the specifics of. On admission, his vital signs included a blood pressure of 120/66 mmHg, a pulse of 125 bpm, a temperature of 97.5^o^F, and an oxygen saturation of 98% on room air. On physical exam, JVD was noted, and moderate rales were auscultated bilaterally. Generalized abdominal tenderness was exhibited with mild guarding in the lower abdomen. While waiting on initial laboratory data, the patient became severely hypotensive and hypoxemic, requiring central line access and rapid intubation.

Initial laboratory data demonstrated a significant array of disturbances. Pertinent lab results included an elevated lactic acid of 4.7mmol/L (normal is <2mmol/L), Brain natriuretic peptide (BNP) of 3,324 pg/ml (normal >100pg/ml), and a high sensitivity troponin of 2,307 ng/L (normal is <14ng/L).

**Figure 1:**
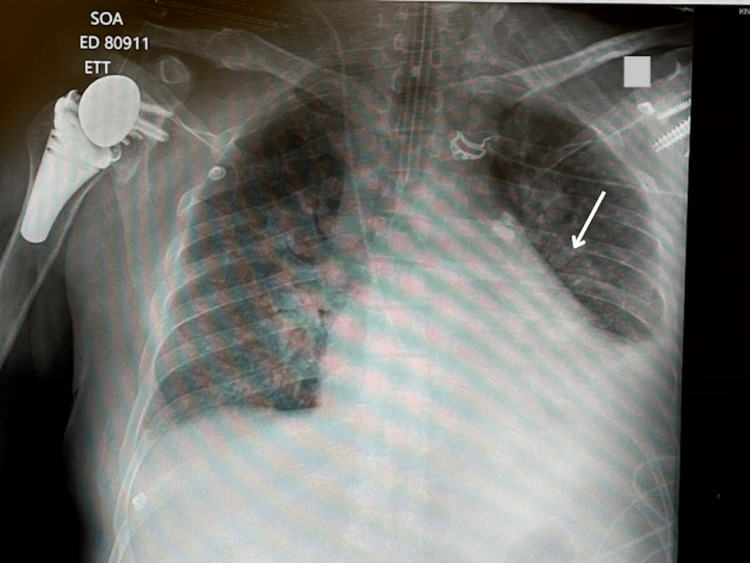
Portable X-ray of the chest demonstrating pleural effusions CXR: Chest x-ray. Arrow displays hazy densities, a fluid collection in the pleural space.

**Figure 2:**
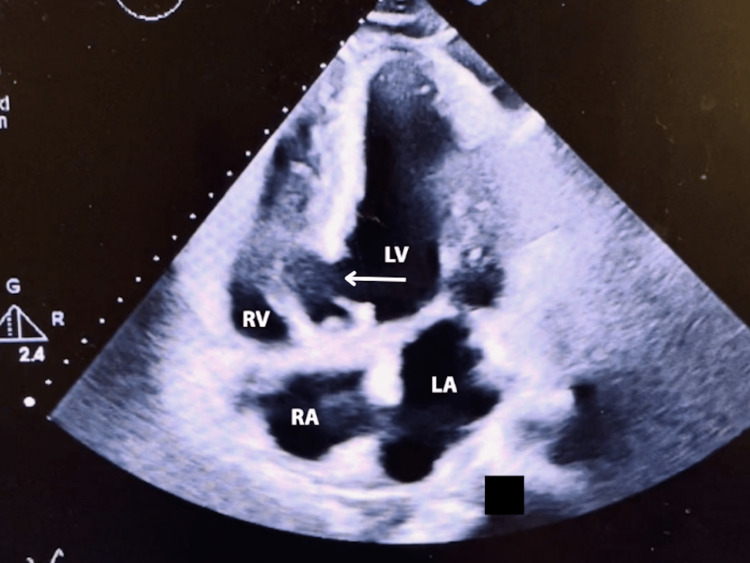
Transthoracic Echocardiogram demonstrating muscular wall defect in the left ventricular septum (arrow)

**Figure 3:**
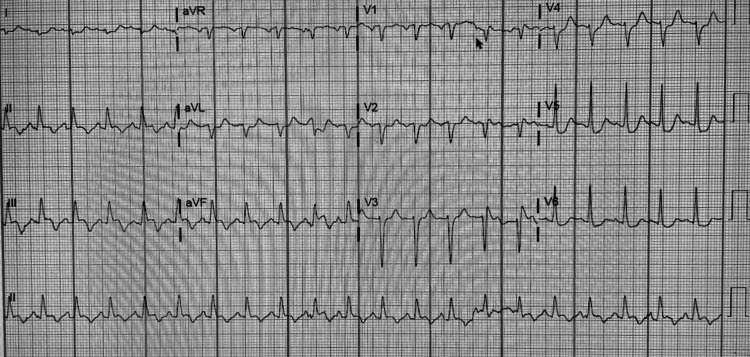
EKG displaying sinus tachycardia, left atrial enlargement and an old anterolateral infarct

Imaging findings demonstrated pleural effusions noted on chest X-ray (Figure 1). A new muscular ventricular septal defect was noted on the transthoracic echocardiogram, along with mitral and mild/moderate tricuspid regurgitation (Figure 2). Additionally, a 12-lead electrocardiogram displayed old anterolateral infarct, sinus tachycardia, and LA enlargement (Figure 3).

All these findings and a rapid hemodynamic instability led to a diagnosis of new-onset ventricular septal defect and acute on chronic systolic heart failure due to left ventricular septal rupture. However, the diagnosis was prolonged because this patient had no previous cardiovascular history, no report of a recent MI, and only just began presenting with congestive symptoms 3 days ago.

Once the diagnosis of ventricular septal rupture was discovered, arrangements for transportation to a different facility were arranged. The original facility where the patient was admitted had no resources to conduct such a highly risky and complicated surgery. However, due to the deadly nature of this complication and our patient's deteriorating condition, our patient required cardiac resuscitation, which was ultimately unsuccessful. 

## Discussion

Left ventricular septal rupture is one of the rare complications that can occur during the first week after a full-thickness myocardial infarction. Once the transmural infarction occurs, areas specific to the blood vessel are compromised and weak. Next, coagulation necrosis occurs, resulting in a loss of blood supply and weakening of the septum leading to eventual rupture. This new connection between the right and left ventricle allows for oxygenated blood from the high-pressure left ventricle to flow to the low-pressure right ventricle causing hemodynamic instability. On top of mixing oxygenation and deoxygenated blood, gradual volume overload in both ventricles leads to pleural effusions and eventual heart failure. Our patient exhibited rapid hemodynamic instability and volume overload symptoms [1].

Management of a left ventricular septal rupture includes surgical repair unless the patient is stable enough to delay surgery. Stable patients have no evidence of cardiogenic shock, minimal or no signs of congestive heart failure, minimal use of vasopressors, no fluid retention, and normal kidney function [2,3]. The reason for a preferred delay in a more stable patient is that the earlier the repair, the higher the mortality since the myocardial tissue is friable and will have difficulty holding the sutures. A delay can facilitate more stable closing as fibrosis kicks in. However, surgical intervention is warranted if the patient is unstable [3,4].

Our patient had no cardiovascular history, no recent MIs noted, and a history of smoking as his only risk factor for a potential post-myocardial infarction mechanical complication. This, along with his generalized symptoms that included signs of cardiovascular congestion and extra-cardiac manifestations such as abdominal pain and urinary retention, delayed the diagnosis of a ventricular septal rupture. Clinical suspicion of a mechanical complication of a silent myocardial infarct was determined only after imaging results, along with the patient's rapid hemodynamic decline, which delayed proper management. By the time diagnosis was made and transfer to a different hospital was arranged, it was too late, highlighting the rapid progression of a VSR and the importance of an early diagnosis.

This case highlights the importance of a high clinical index suspicion of a post-MI mechanical complication in patients with new-onset cardiovascular congestion and extra-cardiac manifestations such as oliguria, constipation, and generalized abdominal pain. This can occur even in patients with no previous cardiovascular history or minimal risk factors. So rapid evaluation should be taken immediately to ensure proper and prompt management.

## Conclusions

While ventricular septal ruptures are rare due to advances in reperfusion therapies, it is crucial to maintain high clinical suspicion and thorough physical examination in a patient presenting with a possible post-infarction complication. Silent myocardial infarctions are not discovered unless patients have diagnostic tests done. Therefore, patients at risk of cardiovascular events must be on the proper medications and regularly managed by their doctor.
